# Bibron’s Antidote

**Published:** 1859-08

**Authors:** D. O. C. Heery

**Affiliations:** Atlanta, Georgia


### ARTICLE IV.

"*Bibron's Antidote.*" By D. O. C. HEERY, M. D., Atlanta, Georgia.

In traveling through south-western Georgia, in April last, I happened at the house of Col. B. Shortly after my arrival, he informed me that one of his most valuable negroes had just been bitten by a large rattlesnake (crotallus confluentus,) while returning from the field. The negro was bitten on the ankle of the left leg. The snake inflicted a very deep wound, and within five minutes after the bite, before much pain or swelling had ensued, I administered one dose of Bibron's Antidote, in two tablespoonful of brandy, and the symptoms almost immediately disappeared. One hour after the bite, pain and swelling returned, attended with considerable throbbing. I repeated the antidote, and in less than fifteen minutes the ankle had regained its natural appearance—all pain and swelling having vanished. Before returning, I repeated the dose a third time. In the morning he was perfectly well, and resumed his duties in the field.

Having read of the wonderful cures performed by Bibron's antidote, I was determined to test its virtue in the first case that I should be called upon to attend. It being generally known that this part of Georgia was numerously infested with these dangerous reptiles, I improved the opportunity, and procured a bottle of Bibron's antidote. I had the good fortune to test its specific qualities, and argue for it a world-wide fame, and fully confirm its claim to the unreserved patronage of the Æsculapian legion. The antidote in question is prepared according to the following recipe, to-wit: "Bibron's Antidote"—℞.—Potassi iodide, grs. iv; hydrarg. chloridi corros., grs. ij; bromini, ʒv.—M. Dose, gtt. x, in two tablespoonsful of brandy, repeated if necessary.

